# Increased Risk of Virologic Rebound in Patients on Antiviral Therapy with a Detectable HIV Load <48 Copies/mL

**DOI:** 10.1371/journal.pone.0050065

**Published:** 2012-11-15

**Authors:** Timothy J. Henrich, Brian R. Wood, Daniel R. Kuritzkes

**Affiliations:** Division of Infectious Diseases, Brigham and Women's Hospital and Harvard Medical School, Boston, Massachusetts, United States of America; McGill University AIDS Centre, Canada

## Abstract

We investigated the independent effects of HIV-1 ”target not detected” measurements versus those that were detectable but below the limit of quantification by Taqman RT-PCR assay on subsequent viral rebound as there are conflicting data regarding the clinical implications of arbitrary or isolated low-level viremia. Cox proportional hazard regression modeling was used to investigate the independent effects of the first HIV-1 load measurement after introduction of the Taqman RT-PCR assay (time-point 0 [T0]), pre-T0 viral loads, CD4 T cell count, race/ethnicity, gender, age and NNRTI use on risk of a confirmed VL >50, >200, >400 and >1000 copies/mL at 22 months follow-up in analyses of all patients and propensity-matched baseline cohorts. 778 patients had a viral load that was either not detected by RT-PCR (N = 596) or detectable, but below the limit of quantification (N = 182) at T0. Detectable viremia, lower T0 CD4 count, decreased age, and having detectable or unknown VL within a year prior to T0 were each associated with viral rebound to >50, >200 and >400 copies/mL. Overall failure rates were low and <5.5% of all patients had confirmed VL >1000 copies/mL. A majority of patients with rebound >200 copies/mL subsequently re-suppressed (28 of 53). A detectable VL <48 copies/mL was independently and significantly associated with subsequent viral rebound, and is cause for clinical concern.

## Introduction

Monitoring the response to antiretroviral therapy relies upon measurements of HIV-1 RNA, with the goal to achieve virologic suppression, defined as a level below the limit of detection of the assay [1]. As assays have become more sensitive, the frequency of detectable HIV-1 RNA at low levels and below the quantifiable range of these tests has become more common but the clinical significance of such results is unclear [2], [3], [4], [5], [6], [7]. In particular, data regarding the clinical implications of a detectable plasma HIV-1 RNA below the quantifiable limit of 50 copies/mL (very low-level viremia, VLLV) are mixed. Two studies have shown a significant association between sporadic VLLV measurements and viral rebound to above 50 or 400 copies/mL [8], [9]. However, two additional studies did not find significant associations between VLLV and subsequent rebound [10], [11]. The methods for quantifying viral loads (VL) differed between these studies, and confounding may have been introduced, as patient characteristics, such as CD4 T cell counts, time of prior virologic control and the use of NNRTI-based regimens differed between baseline comparator groups [8], [10], [11].

These mixed findings leave clinicians with a conundrum when faced with plasma HIV-1 RNA results that fall into the detectable but not quantifiable range: should such a finding prompt a change of therapy, closer monitoring, or no action at all? Further study is warranted to understand fully the clinical implications of VLLV in various populations and in individuals who rebound with higher viral loads. We investigated the independent effects of ”target not detected” measurements versus those that were detectable but below the limit of quantification using the Roche Cobas Taqman RT-PCR assay on risk of virologic rebound in patients followed at two academic medical centers, and described virologic outcomes of patients experiencing rebound.

## Methods

The Partners Healthcare Human Research Committee reviewed and approved this study. The requirement to obtain informed consent from each individual was waived by the institutional review board as the study was limited to review of existing medical records.

Data from electronic medical records of HIV-1-infected patients on treatment at or after the time the Roche Cobas Taqman RT-PCR assay v.1 was introduced into use were collected at two academic medical centers in Boston, Massachusetts. One institution changed from the Versant bDNA assay (limit of detection  = 75 copies/mL) to the Taqman assay in July 2008, and the second institution changed from the Cobas Amplicor assay (limit of detection  = 50 copies/mL) to the Cobas Taqman assay in December, 2009. Patient information was collected at all available time-points following the first viral load (VL) result obtained with the new Taqman assay (time-point 0 [T0]). Information collected included patient demographics, CD4 T-cell count, VL, and antiretroviral regimen, and if all known pre-Taqman VL assays were below the limit of detection (<assay threshold) one year prior to T0. Patients included in the analysis were selected based on the Taqman assay result at T0: those with VL that was detectable but below the limit of quantification (<48 copies/mL; BLQ), and those with VL reported as target not detected (TND). Subjects were included in the analysis only if they had a minimum of two VL measurements after T0.

Baseline characteristics for the TND and BLQ groups were compared using Fisher's exact test for gender and use of non-nucleoside reverse transcriptase inhibitor (NNRTI) therapy, χ^2^ test for race/ethnicity, and Mann-Whitney U test for age and CD4 T-cell counts. Cox proportional hazard regression modeling was used to investigate the independent effects of the first VL measurement and other demographic and laboratory covariates after introduction of the Taqman RT-PCR assay on the risk of virologic rebound. Rebound groups were defined for study purposes as having a confirmed VL >50, >200, >400 or >1000 copies/mL (or a single VL above the cutoff value if this was the last available determination) with follow-up censored at 22 months. A confirmed VL was defined as a result with at least one consecutive measurement greater than the VL threshold value. The study period was determined by the mean length of follow-up and availability of laboratory data. Patient demographics, CD4 T-cell count at T0, NNRTI use, VL group at T0, and known VL < assay threshold 1 year prior to T0 were included in the Cox regression model. The regression was repeated using a viral rebound definition excluding a single VL above the cutoff value at the last available determination. A second hazard analysis was performed to determine the effects of T0 viral load group using a propensity score matched TND comparator cohort (1∶2 BLQ to TND). Propensity scores were calculated by the nearest neighbor method including all study variables as covariates.

Additional clinical information was obtained from the records of patients with confirmed or last VL >200 copies/mL, including whether or not resistance testing was requested because of virologic rebound, resistance test results, changes in antiretroviral drugs at the time of rebound, and whether virologic suppression was re-established (defined as a confirmed or last VL of <50 copies/mL) after initial rebound. All statistical analyses were performed with SPSS vs. 20 and R Essentials.

## Results

A total of 778 patients had a VL that was TND (N = 596) or BLQ (N = 182) at T0. Baseline CD4 cell counts were significantly lower in the BLQ group (Table 1). The propensity analysis incorporated 540 patients (propensity matched TND = 360, BLQ = 182). Propensity matching reduced standardized differences for all covariates to near zero (all between -0.05 to 0.05), with no significant differences between baseline characteristics in the BLQ or matched TND groups (Table 1).

**Table 1:** Baseline (T0) Study Population Characteristics.

	Viral Load at T0		
	Target Not Detected	<48 copies/mL	P
Whole population, N = 778			
Months (median) of follow-up post T0	18.8	20.0	.821
# of VL measurements (median) post T0	5	5	.56
Patients with known VL < assay threshold 1 year prior to T0	354 (59.4)	98 (53.8)	.198
T0 CD4 count (median), cells/mm^3a^	581	500	.003
Age (median years)	48.5	49.0	.431
Gender			
Male	460 (77.2)^b^	152 (83.5)	.079
Female	136 (22.8)	30 (16.5)	
Race/Ethnicity			
White	350 (58.7)	106 (58.2)	.577
Black	133 (22.3)	48 (26.4)	
Hispanic	85 (14.3)	21 (11.5)	
Other/ not reported	28 (4.7)	7 (3.8)	
Antiretroviral Regimen			
NNRTI-based	268 (45.0)	75 (41.2)	.394
Non NNRTI-based^c^	328 (55.0)	107 (58.8)	
Propensity score-matched, N = 540 d			
Months (median) of follow-up post T0	19.8	20.0	.796
# of VL measurements (median) post T0	5	5	.485
Patients with known VL < assay threshold 1 year prior to T0	203 (56.4)	98 (54.4)	.713
T0 CD4 count (median), cells/mm^3^	540	500^e^	.468
Age (median years)	49	49	
Gender			
Male	305 (84.7)	150 (83.3)	.707
Female	55 (15.3)	30 (17.7)	
Race/Ethnicity			
White	224 (62.2)	104 (57.8)	.245
Black	69 (19.2)	48 (26.7)	
Hispanic	51 (14.2)	21 (11.7)	
Other/ not reported	16 (4.4)	7 (3.9)	
Antiretroviral Regimen			
NNRTI-based	151 (41.9)	73 (40.6)	.782
Non NNRTI-based	209 (58.1)	107 (59.4)	

Abbreviations: T0, time of first viral load result using the ultrasensitive Taqman assay (study entry); TND, target not detected; NNRTI, non-nucleoside reverse transcriptase inhibitor.

a 4 missing values, N = 774.

b Number and percent within T0 viral load group.

c Includes protease inhibitor, integrase inhibitor-based and other regimens.

d Includes 180 subjects with VL <48 copies, and 360 subjects with undetectable VL propensity score-matched to the <48 group.

e Mean CD4 counts were identical after propensity matching.

During 22 months of follow-up, 66 patients in the TND group (11.4%) and 60 in the BLQ group (33%) had a confirmed or last VL >50 copies/mL. Thirty-two (5.4%) and 21 (11.5%) of patients in the TND and BLQ groups experienced rebound to a VL >200 copies/mL, whereas 26 (4.4%) and 18 (9.9%), respectively, rebounded to >400 copies/mL. Only 20 of patients in the TND group (3.4%) and 10 in the BLQ group (5.5%) experienced viral rebound >1000 copies/mL. A similar percentage of patients experienced viral rebound >50, >200, >400, and >1000 copies/mL from the propensity-matched TND cohort when compared to the unmatched TND population (12.2%, 5.8%, 4.7%, and 3.9%, respectively).

Table 2 shows results of the Cox proportional hazard regression analysis. Factors including a VL BLQ at T0, lower CD4 count, younger age, and known detectable viral load prior to T0 by the older VL assays were independently associated with a subsequent confirmed VL >50, >200 and >400 copies/mL for all patients. BLQ VL at T0 was also associated with a subsequent confirmed VL >50, >200, and >400 copies/mL in the propensity-matched cohort study. Of note, the clinical laboratory made no distinction between TND and BLQ prior to the Taqman assay roll-out at T0, but clinicians had subsequent access to the BLQ result.

**Table 2:** Multivariable Results from Cox Proportional Hazards Regression Analyses of Predictors of Virologic Rebound.

	Rebound >50 Copies/mL		>200 Copies/mL		>400 Copies/mL		>1000 Copies/mL	
	HR	95% CI	HR	95% CI	HR	95% CI	HR	95% CI
Whole population a								
T0 Viral load group								
Target Not Detected	1.00	…	1.00	…	1.00	…	1.00	…
<48 copies/mL	3.02^b^	2.12–4.30	1.83^b^	1.05–3.19	1.89^b^	1.03–3.47	1.31	0.61–2.84
Known VL < assay threshold 1 year prior to T0	0.46 b	0.32–0.65	0.32^b^	0.18–0.58	0.26^b^	0.13–0.51	0.26^b^	0.11–0.59
T0 CD4 count, per 100 cells/mm^3^ higher	0.88 b	0.82–0.94	0.81^b^	0.72–0.91	0.75^b^	0.66–0.86	0.72^b^	0.60–0.85
Age, per 10 years older	0.81 b	0.66–0.98	0.69^b^	0.0.51–0.94	0.65^b^	0.47–0.90	0.76	0.51–1.13
Male Gender	1.15	0.75–1.79	0.75	0.40–1.40	0.63	0.32–1.23	0.59	0.25–1.38
Race/Ethnicity								
Black	1.42	0.95–2.12	1.83^b^	1.00–3.33	1.24	0.64–2.39	0.77	0.33–1.81
Hispanic	0.89	0.50–1.58	0.58	0.22–1.54	0.21^b^	0.05–0.88	0.13	0.02–1.01
Other/not reported	0.81	0.34–1.92	0.56	0.12–2.51	0.47	0.10–2.13	0.64	0.13–3.05
NNRTI-based ART	0.85	0.59–1.23	0.84	0.49–1.46	0.93	0.51–1.70	0.91	0.44–1.89
Propensity score-matched c								
T0 Viral load group								
Target Not Detected	1.00	…	1.00	…	1.00	…	1.00	…
<48 copies/mL	3.14^b^	2.13–4.65	2.05 b	1.12–3.76	2.16 b	1.12–4.2	1.43	0.63–3.22

Abbreviations: HR, hazard ratio; CI, confidence interval; T0, time of first viral load result using the ultrasensitive Taqman assay (study entry); NNRTI, non-nucleoside reverse transcriptase inhibitor; ART, antiretroviral therapy.

a N = 773 (5 cases with missing demographic or laboratory values).

b Significant association, P<0.05.

c N = 540, including 180 subjects with VL <48 copies, and 360 subjects with undetectable VL propensity score-matched to the <48 group. Only effects of the T0 VL group were compared as propensity score-matching adjusted for baseline differences between cohorts.

Factors including a VL BLQ at T0, lower CD4 count, and known detectable viral load prior to T0 by the older VL assays were independently associated with a subsequent confirmed VL >50, >200 and >400 copies/mL from Cox regression analysis of all patients defining viral rebound as only a confirmed, consecutive VL above the cutoff value. Younger age was also associated with VL>200 and >400 copies/mL (all P<0.05). Only lower CD4 count and known detectable viral load prior to T0 were independently associated with viral rebound >1000 copies/mL.

Of the 53 patients from the entire population who experienced viral rebound to >200 copies/mL, 55% re-suppressed to <50 copies/mL. Of the 24 patients that did not resuppress, 10 had persistent VL >200, 5 had persistent LLVL >50 but <200 and 9 had no further VL info available. Of note, 26% of patients were receiving NNRTI based therapy at the time of viral rebound, and 30% of patients experiencing rebound to >200 copies/mL underwent a change in ART. Interpretable results were obtained from 22 of 34 genotypic resistance tests attempted. Nine of the 22 viral genotypes showed evidence of drug resistance, including 6 with NNRTI resistance. The median viral load at the time of failure for the 9 patients with resistance was higher than for the 13 patients without measurable resistance (1863 versus 45294 copies/mL, P = 0.006). All 9 patients with documented drug resistance changed antiretroviral regimens, of whom 7 subsequently re-suppressed.

## Discussion

A better understanding of the mechanisms and clinical importance of residual viremia is needed as newer, more sensitive assays are implemented. We show that viral load measurements that are detectable but <48 copies/mL independently predicted a higher risk of virologic rebound to >50, >200 and >400 copies/mL but <1000 copies/mL when compared to undetectable viremia (i.e., TND).The strongest predictor of viral rebound was having quantifiable or unknown viral loads within the year prior to rollout of the ultrasensitive Taqman assay; lower CD4 count at baseline was also a strong predictor of rebound. Although power to detect small differences between viral rebound at higher VL copy numbers was lacking, a higher proportion of patients within the T0 VL BLQ group experienced rebound >1000 copies/mL. As a result, detectable but unquantifiable viral load measurements are cause for concern.

Prior studies that examined the relationship between VLLV and subsequent virologic rebound or failure have given mixed results. For example, a single VLLV was associated with subsequent viral rebound to >50 or 400 copies/mL at 12 to18 months in one study [8], but baseline differences between NNRTI usage, CD4 counts and length of undetectable viremia or treatment duration prior to the baseline VL may have introduced bias into the study, even when adjusted for covariates in Cox regression modeling. When our TND group was propensity-matched to the BLQ group by all baseline variables, the significant association between VLLV and virologic rebound remained. Three additional studies using a cutoff of 50 copies/mL yielded conflicting results [9], [10], [11]. One study identified an increased risk of virologic rebound over 12 months when comparing patients with VL <3 copies/mL to those with VL of 3 to 50 copies/mL [9], but two other studies found no significant increase in VLLV and subsequent virological failure [10], [11].

Previous studies that identified significant positive associations between VLLV and virologic rebound utilized the Abbot RealTime PCR assay for VL testing. We identified a similar association when VL testing was performed using the Roche Cobas Taqman assay v.1. A higher frequency of low-level viral blips using the Cobas Taqman v.1 assay compared to other quantification platforms has been described [3], but the Taqman assay v.1 demonstrated similar performance to the Abbot RealTime in a recent, large comparator study [12]. Version 1 of the Taqman assay has been superseded by v.2 [6], but our findings are particularly relevant given that associations between VLLV and viral rebound have now been identified in studies incorporating different VL quantification methods.

A majority of patients (53%) with viral rebound to >200 copies/mL in our study resuppressed, even though less than a third of patients changed ART regimens. A previous study showed that low-level viremia appeared to be transient, with 40% of patients reverting to complete suppression over time [10]. By contrast, patients with persistent low-level viremia >50 copies/mL experience higher rates of virologic failure >1000 copies/mL and immune activation, and may be at risk of developing antiviral resistance [13], [14].

This study was limited by information available from medical record review. Reliable information regarding CD4 T cell count nadirs, duration of ART and suppressed viremia prior to T0 were not available from the medical records, as patients either presented to care already on therapy or had been followed intermittently at facilities outside of our hospital network. As a result, survivor or other bias may have been introduced into the analyses. Our study incorporated the longest follow-up time used in recent studies of VLLV and was unique in the use of propensity score-matching to reduce standardized differences between baseline variables and investigation of viral rebound at higher viral loads (e.g. >1000 copies/mL) [8], [9], [10].

In conclusion, our finding that VLLV is associated with a greater risk of subsequent viral rebound is a cause for concern, but the implications for clinical management are uncertain. Studies in larger cohorts are needed to answer important clinical questions regarding the potential increased costs of more frequent VL monitoring or switching to more expensive or less well-tolerated 2^nd^ and 3^rd^ line regimens that may result from lowering VL cutoffs that define full viral suppression [15], [16].
